# Solitary Fibrous Tumor of the Pleura: A Clinical Case of a Giant Malignant Tumor With Post-surgical Recurrence

**DOI:** 10.7759/cureus.78713

**Published:** 2025-02-07

**Authors:** Boujemaa Razouq, Hamza Azal, Joel Bayem Cedric, Mouhsin Ibba, Hicham Fenane, Yassine Msougar

**Affiliations:** 1 Thoracic Surgery, Mohammed VI University Hospital, Marrakesh, MAR

**Keywords:** case report, immunohistochemistry, mediastinal neoplasms, mediastinal pleura, pleura, solitary fibrous tumor (sft), surgical resection, tumor recurrence

## Abstract

Solitary fibrous tumor of the pleura (SFTP) is a rare mesenchymal tumor, making up a small fraction of primary pleural tumors. It is typically benign but can display malignant features. This case presents a 59-year-old patient with a giant malignant SFTP located in the right posterior inferior mediastinum, which caused significant compression of adjacent structures, including the lung, heart, and esophagus. The patient initially presented with dyspnea, chest pain, and weight loss. Imaging studies revealed a voluminous mass with heterogeneous characteristics, calcifications, and pleural effusion, while biopsy and histopathological analysis confirmed a mesenchymal proliferation consistent with a solitary fibrous tumor. Despite partial resection, due to the tumor’s size and extensive adhesions, complete removal was not feasible. Postoperative histological findings revealed features indicative of malignancy, including a high mitotic index and slight nuclear atypia. The patient declined adjuvant chemotherapy and radiotherapy, and experienced recurrence within four months. Despite the lack of chemosensitivity, the tumor progressed locally, and the patient’s clinical condition worsened. This case highlights the challenges in diagnosing and managing malignant SFTPs, emphasizing the importance of individualized treatment plans and the necessity for long-term follow-up due to the risk of recurrence and poor prognosis following incomplete resection.

## Introduction

A solitary fibrous tumor (SFT) is a rare mesenchymal spindle cell tumor first described by Wagner in 1870 [1]. Klemperer and Rabin further characterized the pathological features of solitary fibrous tumors of the pleura (SFTP) and classified them as an independent disease in 1931 [2]. SFTP constitutes a rare clinical entity, representing approximately 4% of chest tumors [3]. The incidence of SFTP is evenly distributed between genders, with a propensity for presentation in the fifth and sixth decades of life [4]. Most patients with SFTP are asymptomatic until the tumor reaches a significant size. Approximately 20% of SFTP cases are classified as malignant [5]. The optimal treatment for SFTP entails the total resection of primary and locally recurrent disease. Long-term follow-up is crucial due to the potential risk of recurrence. Herein, we report a rare case of a giant SFTP exhibiting malignant behavior that underwent incomplete surgical resection and subsequent recurrence without chemotherapy.

## Case presentation

**Figure 1:**
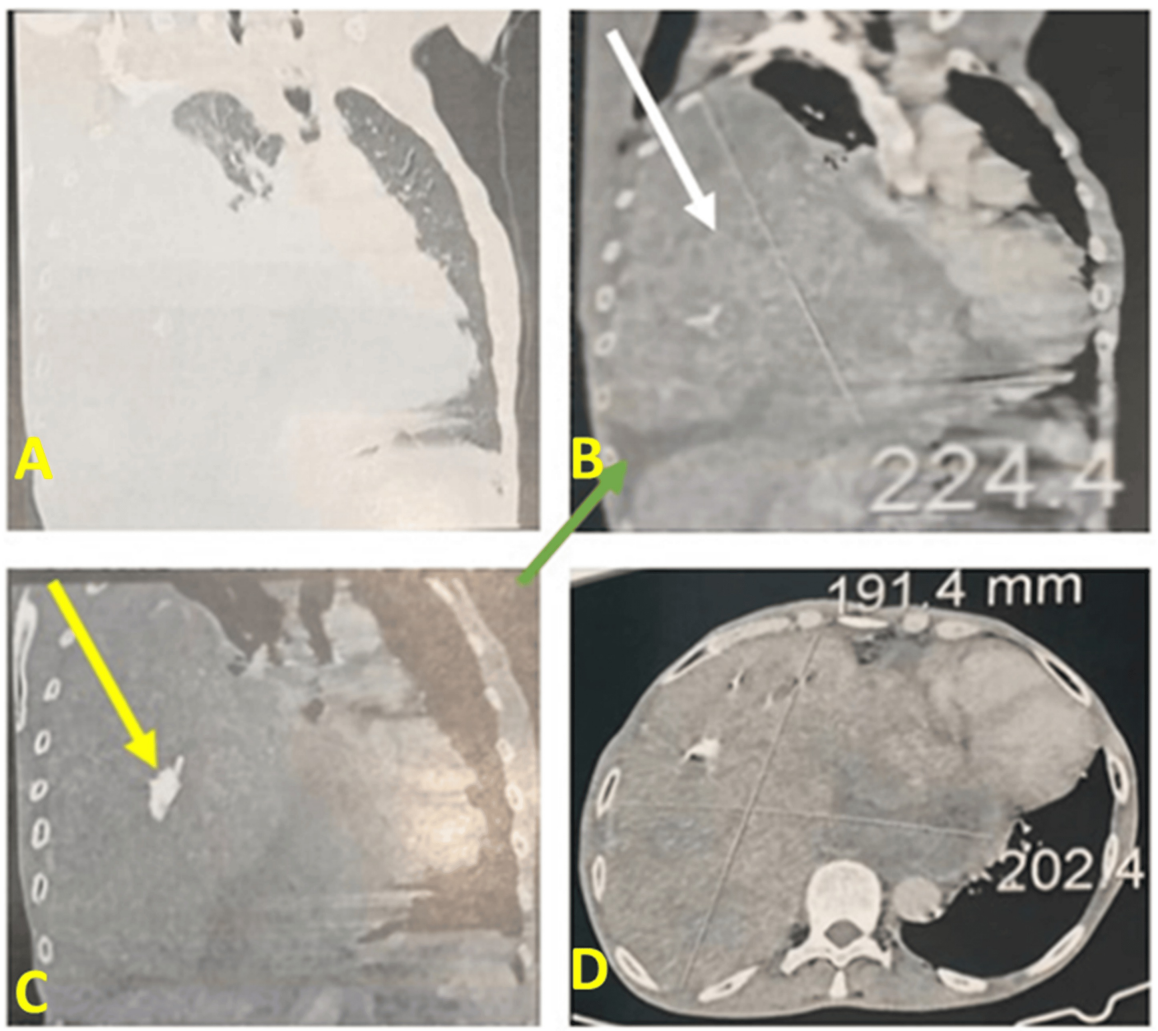
Thoracic computed tomography. (A-D) A large mass (white arrow) in the right posterior inferior mediastinum was measured at 20.2 x 19.1 x 22.4 cm. The mass was heterogeneous in nature, with calcifications (yellow arrow), pleural effusion (green arrow), and mediastinal shift.

This clinical case involves a 59-year-old former smoker with a history of 15 pack-years who ceased smoking 20 years ago. The patient has no documented history of tuberculosis or recent exposure to the infection. Eight years prior, the patient underwent a total thyroidectomy and has since been maintained on levothyroxine at a dosage of 100 µg, with satisfactory follow-up. One month prior to presentation, the patient exhibited dyspnea and right basithoracic pain, without hemoptysis, within the context of a deteriorating general condition characterized by asthenia, anorexia, and a weight loss of 5 kg over the preceding month. The clinical examination indicated that the patient was conscious and demonstrated both hemodynamic and respiratory stability. The pleuropulmonary examination revealed signs consistent with right basal condensation syndrome, while the remainder of the clinical examination was unremarkable. A thoracic CT scan revealed a voluminous right posterior lower mediastinal mass measuring 20.2 x 19.1 x 22.4 cm. The mass exhibited a heterogeneous and solid-cystic composition, with calcifications that were enhanced following iodine contrast injection. It was noted that the mass involved the posterior, middle, and lower mediastinum, resulting in lung displacement, total atelectasis, moderate pleural effusion, and shifting of the left mediastinum (Figure 1).

Abdominal, pelvic, and cerebral computed tomography (CT) scans were determined to be within normal limits. An ultrasound-guided percutaneous transparietal core biopsy was performed. The anatomical-pathological analysis revealed whitish biopsy cores with a firm consistency, which were separately embedded in three cassettes. Microscopic examination demonstrated a proliferation of mesenchymal cells arranged in intersecting bundles. The cellularity was low, with areas of slightly denser cellular aggregates. The cells exhibited a spindle shape, characterized by elongated nuclei and dense chromatin, with no evidence of cytonuclear atypia or abnormal mitotic activity. The cytoplasm was abundantly eosinophilic, while the interstitial tissue was minimal and fibrous, punctuated by clusters of mononuclear inflammatory cells. Blood vessels appeared congested, featuring turgid endothelium and no evidence of hemangiopericytoma. Tumor necrosis was not identified. In conclusion, the morphological characteristics indicate a mesenchymal-like fusocellular proliferation with no definitive signs of malignancy within the material examined.

Immunohistochemistry demonstrated moderate, diffuse tumor cell membrane expression of the anti-CD34 antibody. Furthermore, there was moderate and diffuse nuclear expression of the anti-STAT6 antibody in tumor cells, which is consistent with a diagnosis of OMS2020 solitary fibrous tumor. In conclusion, the morphological and immunohistochemical characteristics of mesenchymal proliferation are predominantly remodeled by hyaline fibrosis within the context of a solitary fibrous tumor (WHO 2020) [6].

**Figure 2:**
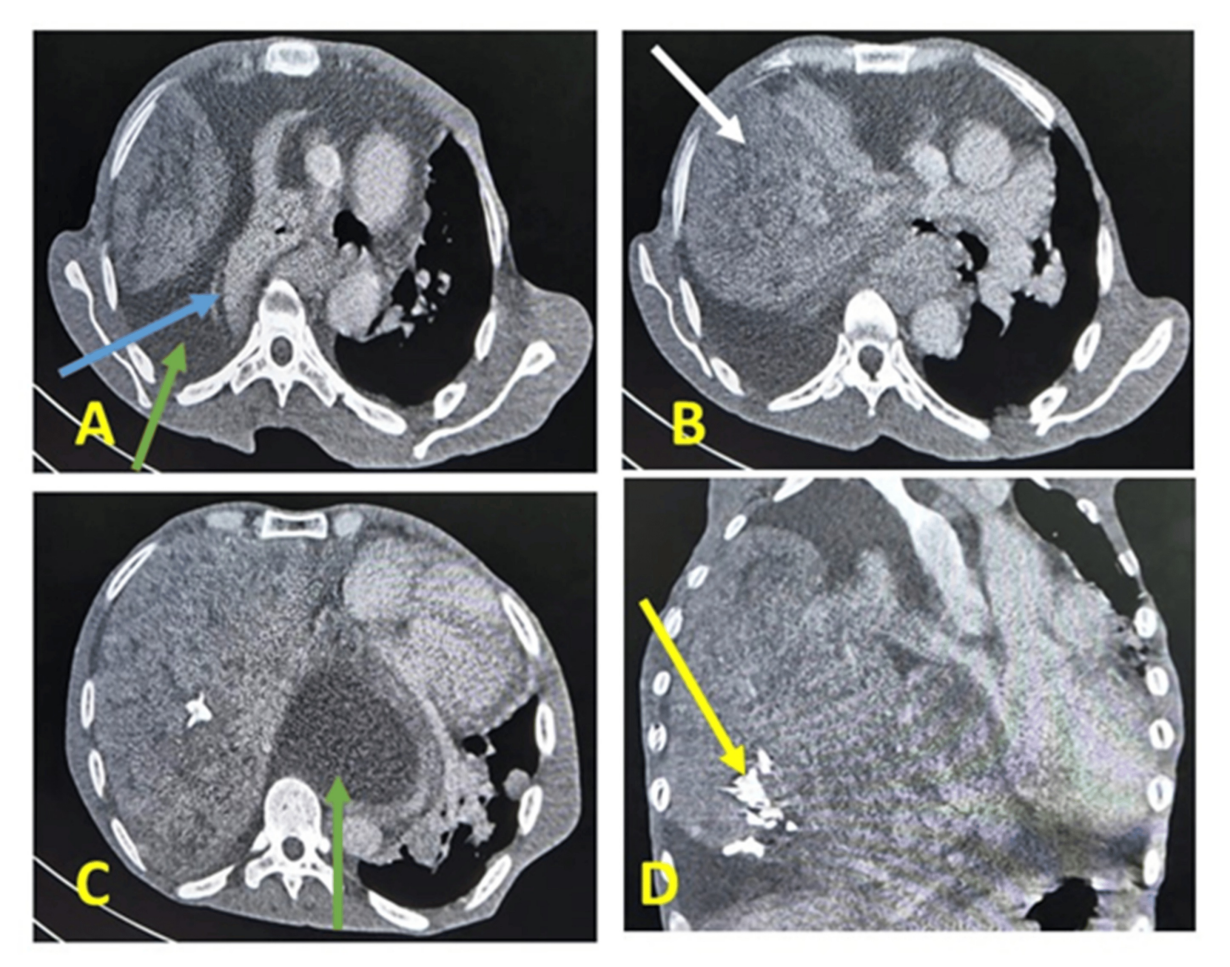
Chest computed tomography after biopsy (A-D) Reveal a worsening pleural effusion (green arrow) around the tumor (white arrow), with calcifications (yellow arrow), left lung atelectasis (blue arrow), and a shift in the mediastinum.

The patient exhibited respiratory instability characterized by severe dyspnea, acute respiratory failure, diaphoresis, and altered consciousness, necessitating transfer to the intensive care unit. The patient underwent multiple sessions of non-invasive ventilation, and an emergency chest CT scan revealed worsening pleural effusion and left mediastinal compression (Figure 2).

Comprehensive biological tests were conducted, including assessments of thyroid hormones, hemoglobin, leukocyte count, renal function, liver function, blood glucose levels, and blood ionogram, all of which yielded normal results.

Tumor markers, including alpha-fetoprotein (AFP), carbohydrate antigen 19-9 (CA 19-9), carcinoembryonic antigen (CEA), human chorionic gonadotropin (HCG), and prostate-specific antigen (PSA), were all found to be within normal limits.

Thoracic drainage revealed a bloody pleural effusion, and the Rivalta test produced a positive result. The lymphocyte and neutrophil counts were 55% and 45%, respectively. Total protein levels were measured at 35.40 g/L, and lactate dehydrogenase (LDH) was recorded at 171 U/L. The adenosine deaminase (ADA) test was not conducted. Cytological analysis of the pleural fluid identified a limited number of mesenchymal cells, with no malignant cells detected.

**Figure 3:**
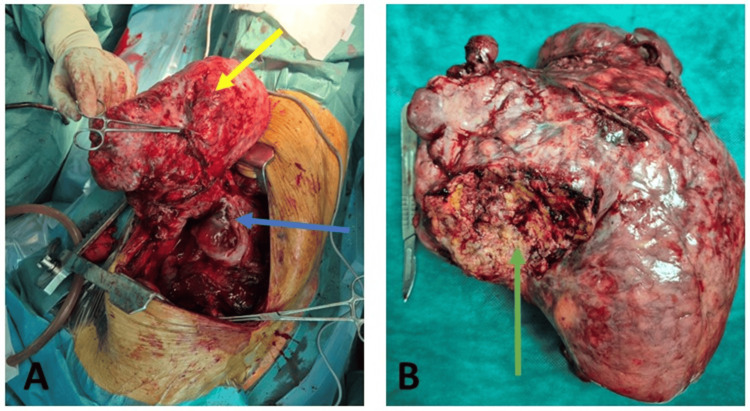
Surgical images. (A) Showing the bulky tumor (yellow arrow) and the tumor residue (blue arrow); (B) The resected tumor with the sectioned area (green arrow).

After thorough preparation, a right posterolateral thoracotomy through the fifth intercostal space was performed. The execution of a total vertical sternotomy and video-assisted thoracoscopy was complicated by the size and location of the mass. Initial exploration revealed a substantial mediastinal mass occupying the entire right hemithorax, accompanied by complete atelectasis of the right lung. The release of adhesions and manipulation of the mass towards the mediastinum facilitated the decompression of mediastinal structures and allowed for bi-pulmonary ventilation, resulting in the re-expansion of the right lung. The mediastinal mass was characterized by a large and firm consistency, exhibiting extensive adhesions and infiltration into adjacent tissues, particularly the mediastinal pleura, inferior vena cava, pulmonary hilum, and heart. Complete monobloc resection was deemed unfeasible, leaving a 4 cm tumor residue in situ, as denoted by surgical clips (Figure 3).

**Figure 4:**
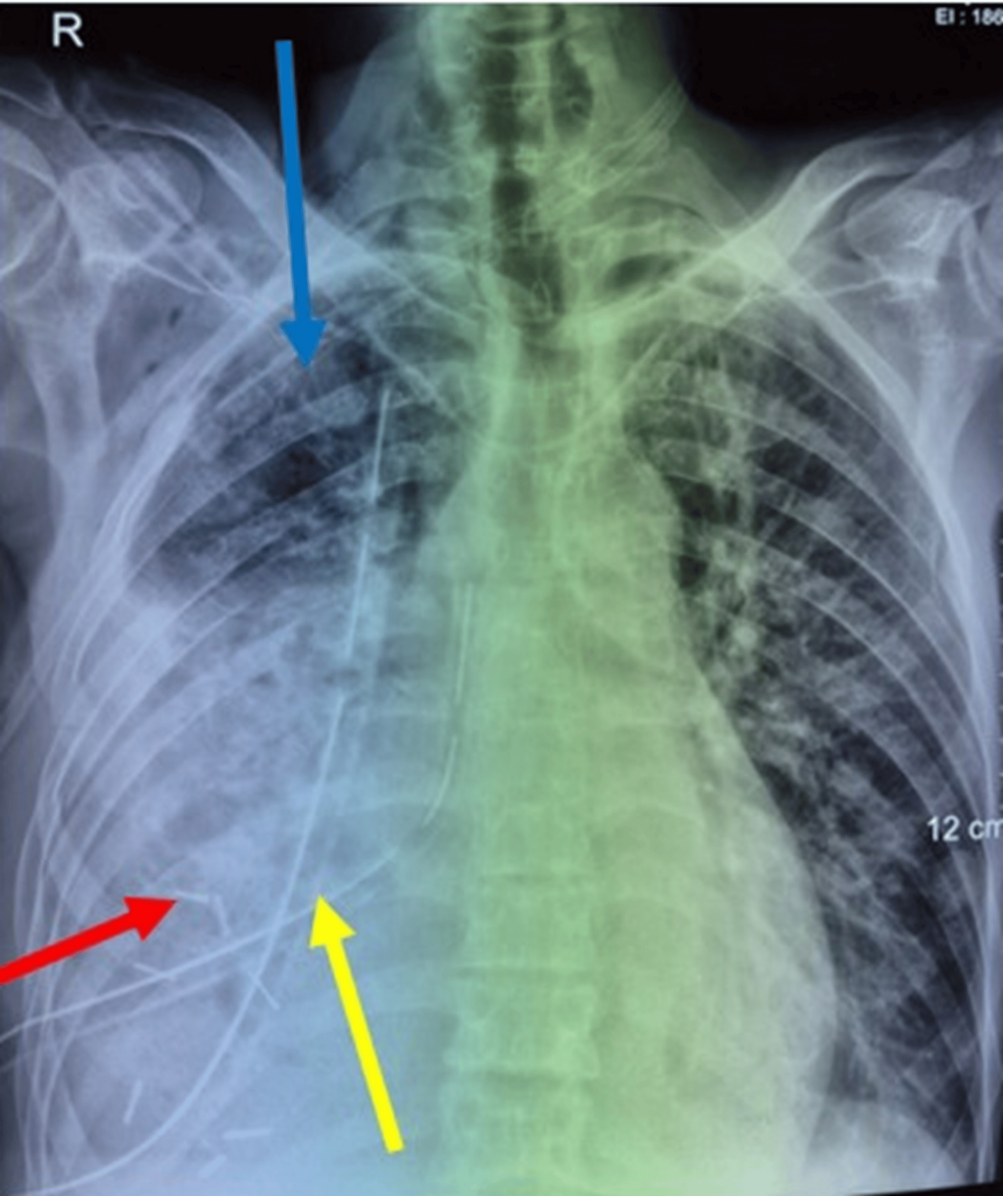
Chest X-ray. Revealed re-expansion of the lung adhered to the wall (blue arrow), with drains in place (yellow arrow) and clips on the tumor residue (red arrow).

The patient demonstrated a positive clinical, biological, and radiological recovery trajectory (Figure 4) and was subsequently transferred to the oncology department for further management.

An anatomopathological examination of the operative specimen revealed an excisional specimen weighing 2800 grams and measuring 28.0 x 20.0 x 11.0 cm. The specimen was encapsulated and well-defined. Upon cross-sectioning, it exhibited a beige coloration, firm consistency, and multinodular appearance alongside areas of hemorrhagic and fibrous remodeling. Microscopic analysis demonstrated hyaline fibrosis in two-thirds of the tissue samples. Additional tissue samples from the periphery of the excision site exhibited tumor proliferation characterized by moderate to high cell density arranged in short, intersecting bundles of epithelioid cells. The tumor cells displayed elongated nuclei, some of which were slightly enlarged and exhibited slight irregular atypia. The mitotic index was estimated at five mitoses per ten high-magnification fields. The cytoplasm was moderately abundant and eosinophilic. The stroma was fibrous and collagenous, frequently exhibiting hyalinization, along with significant hemangiopericytic vascularization. No tumor necrosis was observed. Immunohistochemical analysis revealed moderate diffuse membrane expression of the anti-CD34 antibody in the tumor cells, as well as moderate and diffuse nuclear expression of the anti-STAT6 antibody. There was an absence of expression for anti-cytokeratin, anti-EMA, and anti-CD31. Additionally, moderate and diffuse nuclear expression of anti-Ki67 was noted in 15% of the tumor cells.

**Figure 5:**
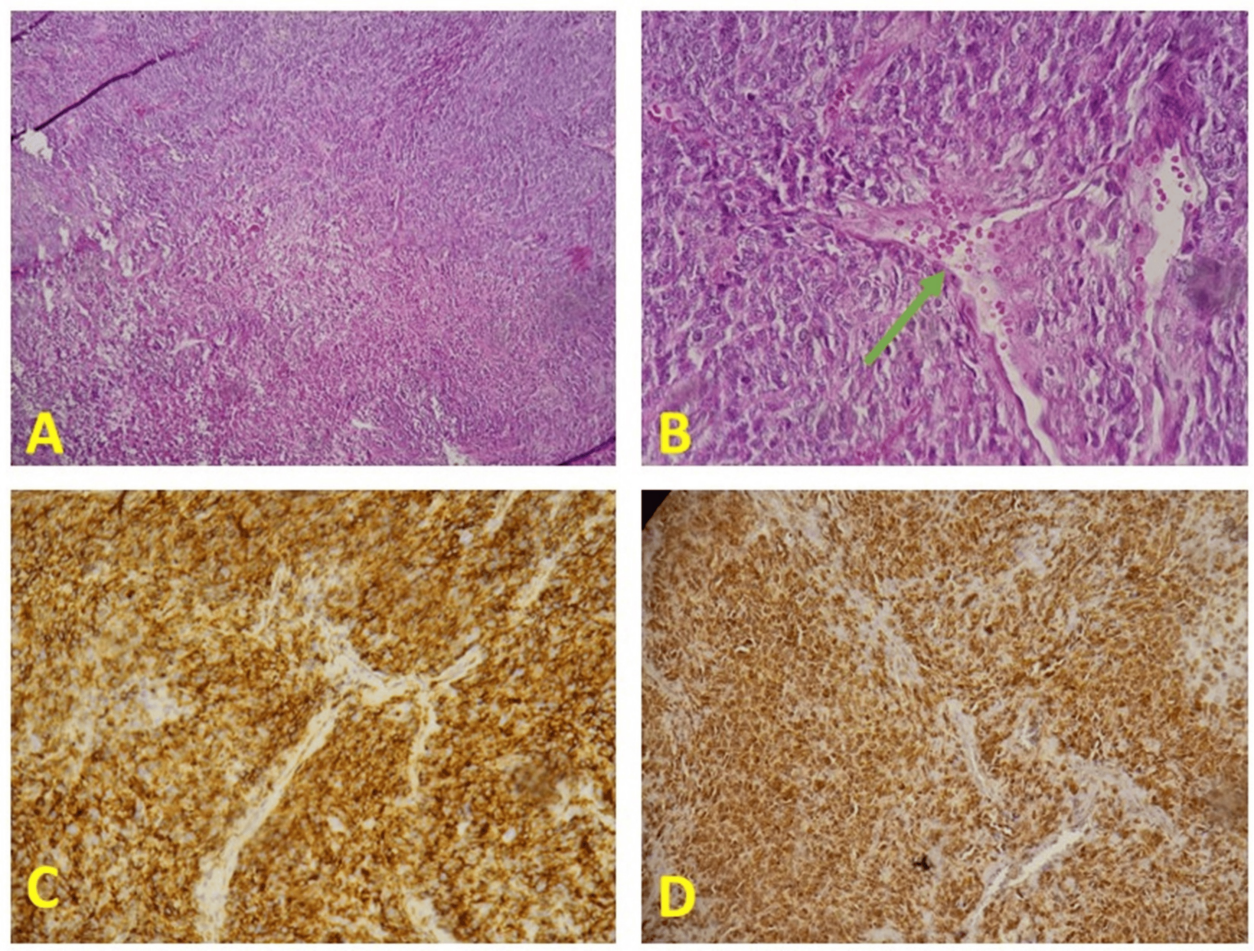
HIstopathological Images. (A) Tumor proliferation with moderate to high cell density made up of epithelioid cells with elongated nuclei and an estimated mitotic index of four mitoses/10 CFG (G×100); (B) Hemangiopericytic vascularization (G×200) (green arrow); (C) Intense, diffuse membrane staining of tumor cells with anti-CD34 antibody (Gx200). (D) Intense, diffuse nuclear staining of tumor cells with anti-STAT6 antibody (Gx200).

Morphological and immunohistochemical analysis demonstrated a mesenchymal proliferation largely modified by hyaline fibrosis, suggesting a solitary fibrous tumor according to the World Health Organization (WHO) 2020 classification [6], with a high risk of recurrence according to the score proposed by the French sarcoma group (age>55 years, size>20.0 cm, five mitoses out of 10 large focal fields, absence of necrosis) (Figure 5).

**Figure 6:**
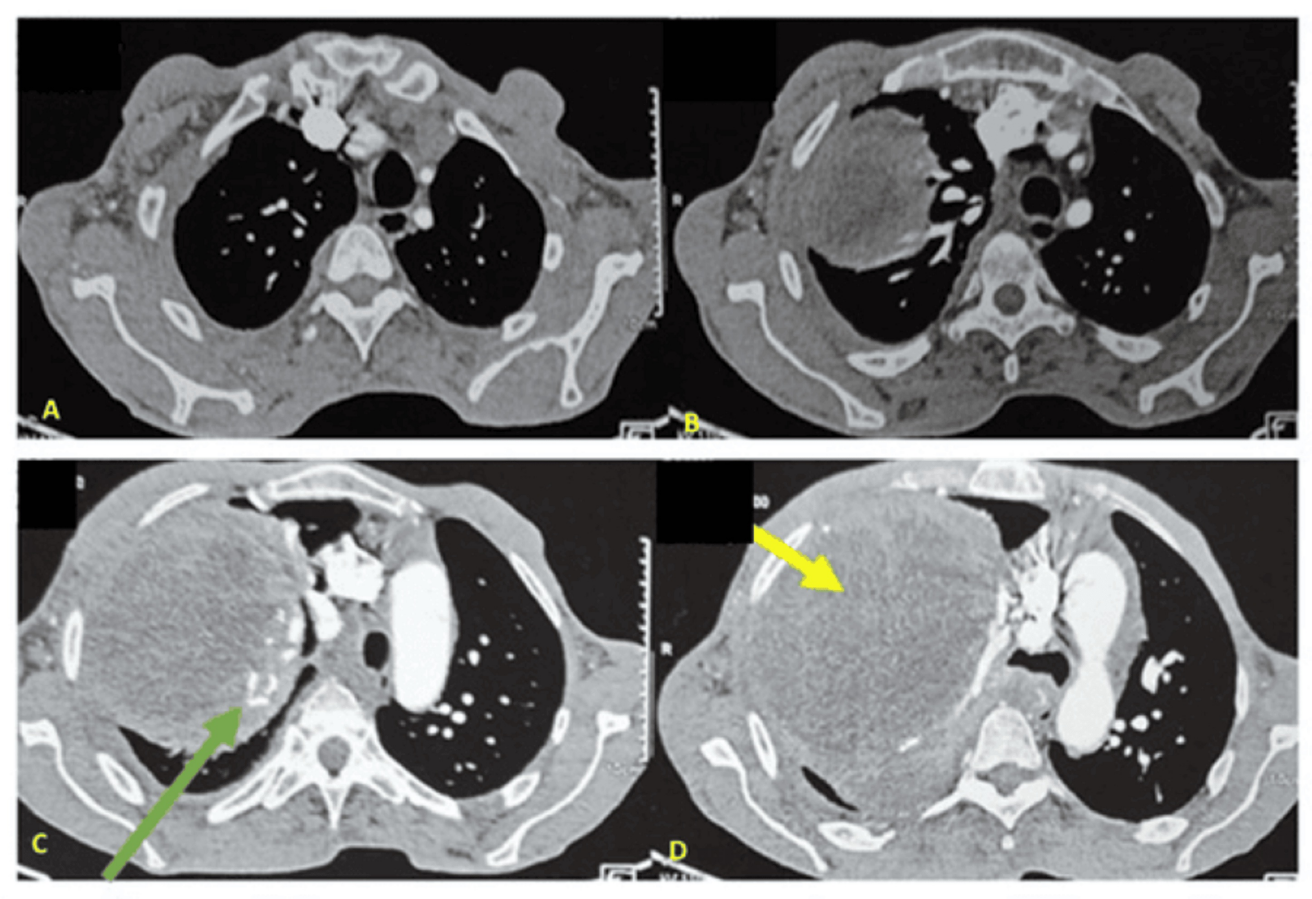
Computed tomography images. (A-D) Voluminous right basithoracic and posterior mediastinal mass (yellow arrow), measuring 184 x 180 mm, heterogeneous with extensive necrosis and calcifications (green arrow) suggestive of tumour recurrence.

The patient was proposed adjuvant chemotherapy and radiotherapy targeting the tumour residue but refused these treatments. Four months after surgery, a deterioration in his clinical condition was observed due to tumor recurrence (Figure 6).

It was decided not to administer palliative chemotherapy due to the low chemosensitivity of the tumor. The disease has progressed locally, and unfortunately, six months after surgery, the patient remains severely weak and malnourished.

## Discussion

SFTP can present in diverse locations, including the mediastinum, pericardium, orbit, peritoneum, intracranial space, kidney, and prostate; nevertheless, the pleura remains the most common site of occurrence [1]. Tumors that measure greater than 15.0 cm or occupy more than 40% of the hemithorax are classified as giant SFTPs [5]. In the present case, the tumor measures 22.0 cm and occupies nearly the entirety of the right hemithorax. SFTs can occur in connective tissue irrespective of age, indicating no significant age-related differences in the onset of SFTs.

A review of 378 cases of SFTP documented in both Chinese and English literature revealed that 195 cases were male and 183 cases were female, with an age range of onset spanning from 6 to 81 years. The disease typically manifests in middle age, exhibiting no notable gender disparity [7]. Intrathoracic solitary fibrous tumors (SFTs) predominantly arise from the pleura; however, these tumors may also originate from the mediastinum. Notably, the anterior mediastinum is the site where the majority of SFTs occur within this region [8]. In contrast, our patient presented with an unusually located, voluminous posteroinferior mediastinal mass, which resulted in compression of the right lung, esophagus, and cardiac chambers. Approximately 80% of SFTP are benign, with more than 50% remaining asymptomatic [5]. However, depending on their size and location, these tumors can present with a diverse range of clinical manifestations, which may include dyspnea and chest pain [8]. It is documented that some patients with SFTP may develop "paraneoplastic syndromes," which can include refractory hypoglycemia, digital clubbing, and pulmonary hypertrophic osteoarthropathy [9]. Furthermore, our patient initially presented with chest pain accompanied by tolerated dyspnea, which subsequently progressed to respiratory instability and impaired consciousness due to respiratory failure secondary to compression. The SFTP combined with pleural effusion is a rare occurrence, with an incidence of less than 20%. Pleural effusion may play a significant role in distinguishing between benign and malignant tumors [10]. Computed tomography (CT) is an essential modality for the clinical diagnosis of SFTP. Benign SFTP typically presents as solitary masses on CT, which are usually round or spindle-shaped, exhibit uneven sizes, have clear boundaries, and demonstrate homogeneous soft tissue density with an intact capsule, either with or without lobulation. The mediastinum and lung parenchyma are often displaced due to pressure. In contrast, malignant SFTP usually presents with unclear boundaries, adhesion to or invasion of surrounding tissues, uneven density, and calcification, and often accompanies pleural effusion [11].

Our case exhibited invasion of the right atrium and ventricle, along with uneven density, calcification, and pleural effusion, all of which are indicative of malignancy. However, in our patient, the pleural effusion may be related to increased exudation caused by tumor compression on the lung and large blood vessels, further aggravated by the biopsy. Despite this, all cytological results of the pleural effusion were negative, which may be due to the complete encapsulation of the tumor, making it difficult for cells to shed into the effusion. Historically, the differential diagnosis for SFTP has been extensive, as the histological differentiation between SFTP and other similar lesions, including mesothelioma, sarcoma, and thymic epithelial neoplasms, has often proven to be unreliable. The characteristic histological appearance of SFTP is characterized by spindle to ovoid-shaped cells arranged in a ‘patternless’ architecture, as identified in the present case. With the advent of immunohistochemical testing, the accurate diagnosis of SFTP has become more feasible; for instance, STAT6 has been recognized as a highly sensitive and nearly perfectly specific marker for SFTP [12].

In this case, we report a rare giant malignant solitary fibrous tumour arising in the mediastinal pleura and showing positive immunohistochemical staining for STAT6. They are most commonly benign lesions; however, malignancy has been identified in as many as 26% of cases. The literature indicates that no single measure of malignant potential is entirely predictive of clinical progression. Nonetheless, a variety of parameters, such as greater than four mitoses per 10 high-power fields, tumor size, nuclear atypia, necrosis, and tumor suppressor gene expression, have been identified as being associated with lesions classified as malignant [13]. In our patient, the tumor demonstrated characteristics associated with a higher risk of malignancy, including a large size (measuring 28.0 x 20.0 x 11.0 cm), a mitotic index above 4 per 10 high-power fields, and slight nuclear atypia. The optimal treatment for SFTs is total resection of both the primary tumor and any local recurrent disease. Patients with SFTs who have undergone surgical intervention exhibit improved overall survival compared to those who have not [14]. Video-assisted thoracoscopic surgery is primarily employed for the resection of small tumors; however, it has also been successfully utilized for the resection of larger fibromas measuring approximately 10 cm [15].

Furthermore, the Da Vinci Surgical System has been reported as effective for the complete surgical resection of an anterior mediastinal tumor in an obese patient. In patients with large tumors or extensive adhesions, thoracotomy is frequently employed, and in instances of incomplete resection or recurrence, secondary surgical intervention may be warranted [16]. In the present case, due to the size of the tumor, minimally invasive surgery was not feasible, and the lesion was excised via posterolateral thoracotomy, which offered better access than median sternotomy. Hemiclamshell thoracotomy was only considered if thoracotomy failed to provide adequate exposure. Adjuvant chemotherapy and radiation were proposed for unresectable tumor residue, but the patient declined due to the tumor's lack of chemosensitivity. Four months later, the patient experienced clinical deterioration due to tumor recurrence, and the patient is now severely weakened and malnourished six months postoperatively.

## Conclusions

This case highlights the rarity and complexity of managing a malignant solitary fibrous tumor (SFT) of the pleura. Despite the partial surgical resection performed, tumor recurrence occurred within four months. The tumor demonstrated characteristics associated with a higher risk of malignancy, including large size, a mitotic index above four per 10 high-power fields, and slight nuclear atypia. The patient, who declined adjuvant chemotherapy and radiation therapy, faced significant clinical deterioration and local disease progression. This case underscores the challenges in treating malignant SFTs, especially in cases of incomplete resection and resistance to chemotherapy. Long-term follow-up and individualized treatment strategies are crucial for improving patient outcomes in such rare and complex cases.
